# Re-annotation of 191 developmental and epileptic encephalopathy-associated genes unmasks de novo variants in *SCN1A*

**DOI:** 10.1038/s41525-019-0106-7

**Published:** 2019-12-02

**Authors:** Charles A. Steward, Jolien Roovers, Marie-Marthe Suner, Jose M. Gonzalez, Barbara Uszczynska-Ratajczak, Dmitri Pervouchine, Stephen Fitzgerald, Margarida Viola, Hannah Stamberger, Fadi F. Hamdan, Berten Ceulemans, Patricia Leroy, Caroline Nava, Anne Lepine, Electra Tapanari, Don Keiller, Stephen Abbs, Alba Sanchis-Juan, Detelina Grozeva, Anthony S. Rogers, Mark Diekhans, Roderic Guigó, Robert Petryszak, Berge A. Minassian, Gianpiero Cavalleri, Dimitrios Vitsios, Slavé Petrovski, Jennifer Harrow, Paul Flicek, F. Lucy Raymond, Nicholas J. Lench, Peter De Jonghe, Jonathan M. Mudge, Sarah Weckhuysen, Sanjay M. Sisodiya, Adam Frankish

**Affiliations:** 1 Congenica Ltd, Wellcome Genome Campus, Hinxton, Cambridge, CB10 1DR UK; 2 Wellcome Sanger Institute, Wellcome Genome Campus, Hinxton, Cambridge, CB10 1SA UK; 3 Neurogenetics Group, Center for Molecular Neurology, University of Antwerp, Antwerp, Belgium; 4 Laboratory of Neurogenetics, Institute Born-Bunge, University of Antwerp, Antwerp, Belgium; 5 European Molecular Biology Laboratory, European Bioinformatics Institute, EMBL-EBI, Wellcome Genome Campus, Hinxton, Cambridge, CB10 1SD UK; 6 Centre for Genomic Regulation (CRG), Barcelona Institute of Science and Technology, Dr. Aiguader 88, 08003 Barcelona, Spain; 7 Universitat Pompeu Fabra (UPF), Barcelona, Spain; 8 Centre of New Technologies, University of Warsaw, Warsaw, Poland; 9 Skolkovo Institute for Science and Technology 3 Nobel St., Skolkovo Innovation Centre, Moscow, Russia; 10 Department of Neurology, University Hospital Antwerp, Antwerp, Belgium; 11 Molecular Diagnostic Laboratory and Division of Medical Genetics, Department of Pediatrics, CHU Sainte-Justine, Montreal, H3T 1C5 Canada; 12 Department of Pediatric Neurology, University Hospital Antwerp, Antwerp, Belgium; 13 Department of Neuropediatrics, CHR Citadelle, CHU Sart-Tilman, Liège, Belgium; 14 Department of Genetics, La Pitié-Salpêtrière Hospital, Assistance Publique-Hôpitaux de Paris, Paris, France; 15 Sorbonne Universities, UPMC Univ Paris 06, UMR S 1127, Inserm U 1127, CNRS UMR 7225, ICM, Paris, France; 16 Pediatric Neurology Department, Timone Hospital, APHM, Marseille, France; 17 Anglia Ruskin University, Cambridge, CB1 1PT UK; 18 Department of Clinical Genetics, Cambridge University Hospitals NHS Foundation Trust, Cambridge, CB2 0QQ UK; 19 Department of Haematology, University of Cambridge, NHS Blood and Transplant Centre, Cambridge, CB2 0PT UK; 20 Department of Medical Genetics, Cambridge Institute for Medical Research, University of Cambridge, Cambridge, CB2 0XY UK; 21 Center for Biomolecular Science and Engineering, University of California, Santa Cruz, CA USA; 22 The Hospital for Sick Children, Toronto, Canada; 23 Department of Pediatrics (Neurology), University of Texas Southwestern, Dallas, Texas USA; 24 The FutureNeuro Research Centre, Royal College of Surgeons in Ireland, Dublin, Ireland; 25 Centre for Genomics Research, Precision Medicine and Genomics, IMED Biotech Unit, AstraZeneca, Cambridge, CB2 0AA UK; 26 Illumina Inc, Great Chesterford, Essex, CB10 1XL UK; 27 North East Thames Regional Genetics Service, Great Ormond Street Hospital for Children NHS Foundation Trust, London, UK; 28 Department of Clinical and Experimental Epilepsy, UCL Queen Square Institute of Neurology, London, WC1N 3BG UK; 29 Chalfont Centre for Epilepsy, Bucks, SL9 0RJ UK

**Keywords:** Molecular medicine, Medical genomics

## Abstract

The developmental and epileptic encephalopathies (DEE) are a group of rare, severe neurodevelopmental disorders, where even the most thorough sequencing studies leave 60–65% of patients without a molecular diagnosis. Here, we explore the incompleteness of transcript models used for exome and genome analysis as one potential explanation for a lack of current diagnoses. Therefore, we have updated the GENCODE gene annotation for 191 epilepsy-associated genes, using human brain-derived transcriptomic libraries and other data to build 3,550 putative transcript models. Our annotations increase the transcriptional ‘footprint’ of these genes by over 674 kb. Using *SCN1A* as a case study, due to its close phenotype/genotype correlation with Dravet syndrome, we screened 122 people with Dravet syndrome or a similar phenotype with a panel of exon sequences representing eight established genes and identified two de novo *SCN1A* variants that now - through improved gene annotation - are ascribed to residing among our exons. These two (from 122 screened people, 1.6%) molecular diagnoses carry significant clinical implications. Furthermore, we identified a previously classified *SCN1A* intronic Dravet syndrome-associated variant that now lies within a deeply conserved exon. Our findings illustrate the potential gains of thorough gene annotation in improving diagnostic yields for genetic disorders.

## Introduction

The developmental and epileptic encephalopathies (DEE) are a heterogeneous group of rare neurodevelopmental disorders, characterised by (a) early-onset seizures that are often intractable, (b) electroencephalographic abnormalities, (c) developmental delay or regression and (d) in some cases, early death.^1,2^ One of the most well-characterised DEEs is Dravet syndrome (DS), previously known as Severe Myoclonic Encephalopathy of Infancy (SMEI). DS presents as prolonged febrile seizures within the first year of life in an otherwise healthy child, evolving into intractable febrile and afebrile seizures with developmental plateauing or regression in the next few years. DS is genetically one of the most homogeneous DEEs, with more than 80% of people shown to carry a de novo SCN1A variant.^3^ Large-scale international research efforts such as Epi25 <http://epi-25.org/>, the Deciphering Developmental Disorders (DDD) study^4^ and the UK 100,000 Genomes Project^5^ are now concentrating on diagnosing people and identifying genes involved in rare disorders including DEE, using chromosomal microarrays, whole exome sequencing (WES) and whole genome sequencing (WGS). However, while numerous genes associated with other forms of DEE are being uncovered, between 60–65% of people remain without a molecular diagnosis.^6,7^

The ability to infer clinical information about a patient’s genome, relies upon reference data sets that help to make informed decisions about putative causative variants. Therefore, the confidence of such decisions is dependent upon the reliability of the underlying data against which a patient’s genome is analysed. For example, the human genome reference sequence is still incomplete, which is demonstrated by the work that the Genome Reference Consortium (GRC) is undertaking to fill remaining sequence gaps, as well as representing different population haplotypes.^8^ However, the incompleteness of the human transcriptome should also be a consideration for genome interpretation, since a complete set of transcripts from all the different tissue types and developmental stages that are naturally present is not yet available. Until it is possible to confidently generate a de novo genome assembly for a patient, in conjunction with a complete transcriptome (and for example, proteome), researchers and clinicians must rely upon data that is available in public databases.

Therefore, improvements in diagnostics can, in part, be achieved through improvements in gene annotation. At the current time, most GENCODE (i.e. Ensembl)^9^ and RefSeq^10^ gene annotations are based on cDNA and EST libraries produced alongside the initial experimental phase of the Human Genome Project, while the large datasets produced by more recent RNA-Seq and long-read sequencing-based projects remain largely unincorporated. Such datasets have the potential to add exons via transcript models to existing gene annotations and these features can provide new insights into genetic disease. For example, ‘expanded’ exomes offer the potential to capture additional disease-linked variants beyond the reach of previous studies. Additional sequences can be added to existing exome ‘panels’ used for diagnostics in the clinic and used to select regions for resequencing in people without a molecular genetic diagnosis. Indeed, this has already been demonstrated for *DLG2*, where newly identified exons were observed to be deleted in people with neurodevelopmental disorders.^11^ Furthermore, the resequencing of annotated regions identified previously missed pathogenic variants linked to epilepsy in *SCN8A*.^12^ Meanwhile, additional transcript features can also be used to reappraise existing variation datasets; providing, for example, new mechanistic explanations for known disease-associated variants and also allowing for the reconsideration of variants from whole-genome studies that had previously been de-prioritised due to an apparent lack of transcription.

In fact, modern transcriptomics datasets have the potential to add an enormous number of transcribed features to gene annotation catalogues and the exact proportion of this ‘transcriptional complexity’ that is linked to gene function and therefore to phenotype, remains hard to fathom. If a portion of observed transcription events for a given gene lack functional relevance, then expanded gene annotations could unknowingly be a source of misleading variant interpretations. In practice, transcript functionality is confirmed in the laboratory, although important insights can be gained from bioinformatics. Evolutionary conservation has long been regarded as a strong proxy for functionality, for example in the observation that coding sequences (CDS) have been subjected to purifying selection or that splice sites are constrained and consistently transcribed in multiple species.^13^ As such, conservation metrics are commonly used in transcript interpretation and thus, variant prioritisation. However, variants linked to poorly conserved transcript features can also be drivers of genetic disease. In particular, it is now well established that many genes utilise alternative splicing to reduce their translational output, redirecting transcription into non-coding pathways via the incorporation of ‘poison exons’.^14^ Here, we classify poison exons as those whose inclusivity in the transcript cause a CDS change (i.e a frameshift and/or premature termination codon (PTC)) that is predicted to trigger the Nonsense-Mediated Decay (NMD) degradation pathway. While some poison exons are ancient, the mode and tempo of regulatory splicing evolution in general remains poorly understood.^15–17^ Alternatively, several recent reports have demonstrated that gene output can be compromised by variants that improve the splicing efficacy of poorly conserved transcription events at the expense of the ‘canonical’ mRNA, i.e. according to a ‘gain of function’ model.^18^

Here, we explore the potential of expanded gene annotation to improve the diagnostics of epilepsy, utilising a manual annotation workflow that is initially agnostic with regards to the potential functionality of these transcript sequences. Overall, this work has added 3550 GENCODE transcript models to 191 genes associated with DEE via the utilisation of publicly available short- and long-read transcriptomics datasets. Subsequently, we create an expanded exome panel for eight genes associated with DS, incorporating 125 transcript regions and after resequencing 122 people with a clinical diagnosis of DS or a similar phenotype, discover two de novo variants within *SCN1A* exonic sequences. Both variants are found within presumptive poison exons that exhibit poor evolutionary conservation. In contrast, we also demonstrate that a third DS-associated variant, previously considered to be intronic and of unknown significance, is present in an alternative *SCN1A* poison exon that has deep conservation. Although further work is required to understand the biological implications of the transcriptional complexity associated within *SCN1A* and the larger set of 191 genes, our findings relating to DS show that from the current bioinformatics perspective, uncertainties regarding transcript functionality are not necessarily a barrier to the utility of these transcripts in disease genetics.

## Results

### Manual re-annotation substantially increases the number of transcripts

We manually re-annotated 191 genes associated with epilepsy as part of the GENCODE project, primarily using publicly available long-read transcriptional datasets including brain-derived SLR-Seq and PacBio Iso-Seq reads (see Methods). In total, 3550 alternatively spliced transcripts were annotated across the 191 genes, increasing the number of transcripts for these genes from 1807 to 5357. All transcripts are included in GENCODE v28; contemporary RefSeq annotation contains 1397 NM and XM transcripts. The majority of added transcripts contain either complete exons (37%) or alternative splice sites within existing exons (21%). In total, more than 674 kilobases (kb) of additional exonic sequence was added across the 191 genes.

**Fig. 1:**
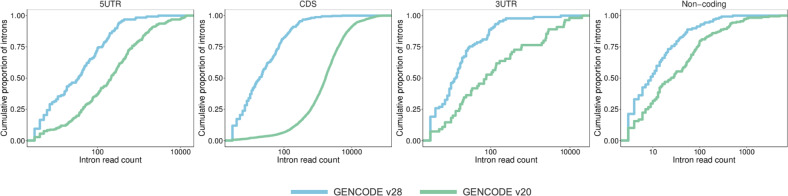
Expression of transcripts. Cumulative distribution curves for the number of intron-supporting reads in pre-existing (GENCODE v20) versus updated (GENCODE v28) annotation. Distribution curves for overall transcripts, CDS, 5′ and 3′ UTR are given. The *x*-axis is in log10 scale.

Next, given the epilepsy context of this study, we further characterised the transcript regions using existing RNA-Seq data from pre-frontal cortex in 36 individuals across 6 life stages.^19^ Introns are generally less well supported than pre-existing introns (Fig. 1) and just 19% of introns between exons annotated as coding are covered by at least 10 RNA-Seq reads. However, while most detectable introns displayed a broadly ubiquitous temporal expression pattern, a subset of 208 (8%) showed a fivefold enrichment in expression in foetal versus infant pre-frontal cortex samples, with 101 showing more than fivefold higher expression than all other samples combined. This enrichment may highlight a subset of transcripts of particular functional importance. However, we recognise that the utility of tissue-specific expression patterns to infer functionality remains debatable.^20,21^

Evolutionary conservation is a commonly used metric of functional potential.^22^ Firstly, we mapped all human annotation for these 191 genes to the mouse reference genome using TransMap.^23^ In all, 40% of introns mapped to the mouse genome with the preservation of canonical splice sites, compared to 87% of introns in the pre-existing annotation, indicating that the annotated introns show generally lower conservation. Secondly, we examined whether there was a correlation between general evolutionary conservation and the decision to annotate a transcription event as coding. We found that 12.7% of the additional exonic coverage in the annotation overlaps with PhastCons elements,^24^ i.e. regions of the genome exhibiting detectable sequence conservation, compared to 47% of pre-existing exons. We next, randomly reassigned the sequences across the genome sequence in order to obtain a background estimate of conservation, finding that the resulting distribution is centred around a 5% overlap following 1000 replicates (not shown) and a one-sample *t*-test supports significant enrichment in the annotations with *p* < 2.2e^−16^. When considering only the 42 kb of sequence annotated as coding, this proportion rises to 23%. However, just 6% of all CDS exhibits the characteristic pattern of protein sequence evolution, as judged by an examination of overlap with regions of positive PhyloCSF score,^25^ compared to 91% of pre-existing coding exons. This may suggest that our annotation has significantly overestimated the proportion of the transcribed sequence that is translated. Alternatively, it could be that certain identified CDS regions have arisen in the primate lineage following the divergence from the rodent clade.

In summary, while expression- and conservation-based approaches do not provide vigorous support for the existence of widespread functionality across the transcribed regions, they do suggest that the reannotation of 191 genes has added a modest subset of models with conserved biological roles.

### Updated annotation identifies putative clinically relevant variants

**Fig. 2:**
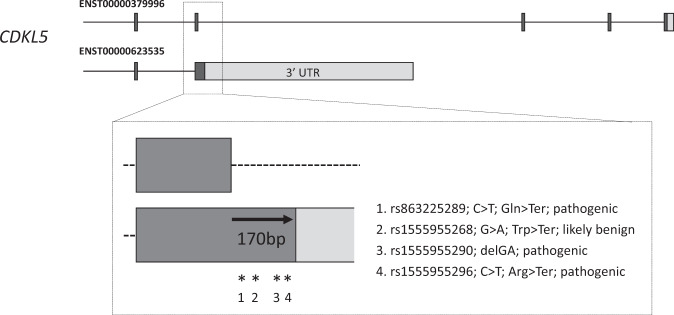
Variants in coding sequence of *CDKL5*. ENST00000379996 had previously been annotated in GENCODE and represents a known protein-coding transcript; coding exons 17–20 are shown (coding exons in black; UTR in grey). ENST00000623535 was annotated as part of this study and the transcript contains an alternative CDS based on the usage of a different C-terminus, linked to a 3′ UTR sequence that extends into an intron of ENST00000379996. This alternative 3′ UTR has strong support in polyAseq experiments and RNA-Seq assays across multiple tissues (not shown). The 170 bp of CDS added to the intronic region contains 4 ClinVar variants, listed here by their dbSNP I.D. alongside the consequences as presented by ClinVar. Bodian et al. recently reported rs1555955290 as a de novo frameshift variant in a child with early onset seizures. Variants rs1555955296 and rs863225289 are de novo nonsense variants submitted to ClinVar by private testing laboratories, both from people described as having early infantile epileptic encephalopathy 2. In contrast, nonsense variant rs1555955268 is currently classified as ‘likely benign’ by ClinVar; this is a privately submitted germline variant from an individual with an unspecified condition. Additional transcript models within the gene have been omitted for clarity.

Given these considerations, we decided to investigate the clinical impact of the annotations without initial regard to their expression or conservation metrics. First, we compared the overlap of pre-existing and updated annotation with the public variation dataset in ClinVar <https://www.ncbi.nlm.nih.gov/clinvar/>. We note that ClinVar is currently heavily biased towards ‘known’ gene sequences in terms of content, i.e. disease-associated variants are less likely to be found in regions that are less well studied (or have been resequenced less often). Nonetheless, we found that 23 existing ClinVar variants could be made ‘exonic’ by our annotations (Supplementary Data 1, sheet 1). This set does not include a further 36 ClinVar variants that fall within 8 bp of an existing splice site, due to the possibility that any pathogenicity associated with these variants is due to the disruption of splicing and not directly associated with the annotation (Supplementary Data 1, sheet 2). Of immediate importance, within the 23 variants are rs1555955290, rs1555955296 and rs1555955268, located within close proximity in CDS added to *CDKL5* (see Fig. 2). The first variant (rs1555955290) is a de novo variant recently identified in a child with early onset seizures.^26^ This variant was recognised by the authors as a 1-bp deletion in the CDS annotation based on RefSeq coding model NM_001323289.1, which was created after the annotation produced by our study was publicly released. Variant rs1555955296 is also a de novo variant classed as pathogenic by ClinVar, found in a patient classified as having early infantile epileptic encephalopathy 2 by a clinical testing laboratory. Interestingly, rs1555955268 is currently classified as ‘likely benign’ by ClinVar; this is a privately submitted germline variant from an individual with an unspecified condition. Nonetheless, like rs1555955296 which is 109 bp downstream, it is also a nonsense variant. It would therefore be surprising if rs1555955268 does not also turn out to have disease associations, although this remains to be ascertained. Finally, we observe that a fourth ClinVar variant (rs863225289) was initially filtered out as it is found 3 bp downstream of the pre-existing splice donor site. This variant was also provided to ClinVar by a clinical testing laboratory, being found in a patient with early infantile epileptic encephalopathy 2. While the variant has been classed as pathogenic, it remains unconfirmed whether the inferred causal effect is due to splicing disruption or loss of function in the CDS.

**Fig. 3:**
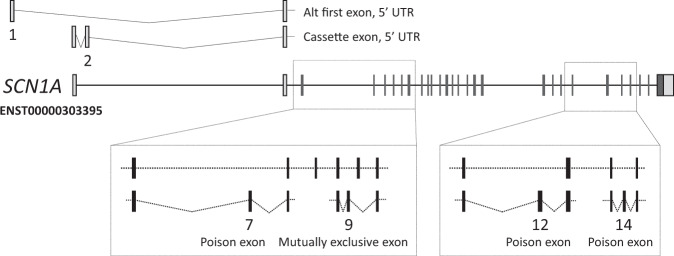
The updated *SCN1A* annotation identified 10 exons and five shifted splice junctions, increasing the genomic footprint of *SCN1A* transcription by ~3 kb. All features are described with respect to existing Ensembl model ENST00000303395 and numbered according to the scheme used in Table 1. For clarity, the features are shown as truncated models containing only the exons of specific interest (and certain features are present on multiple transcript models in the complete gene annotation). UTR sequences are shown in grey, coding or NMD regions in black. Features [1] and [2] represent previously unreported 5′ UTR sequences that have conservation and equivalent expression in mouse and chicken. Features [7] and [14] are cassette exons predicted to invoke NMD and contain the de novo variants identified in the study within patients one and two respectively. Feature 9 is a cassette exon that is an ancient duplication of coding exon five, to which it is transcribed in a mutually exclusive manner; the clinical significance of this exon has been previously demonstrated by Tate et al. Feature 12 is a cassette exon predicted to invoke NMD. Intron and exon sizes are to approximate scale. Additional transcript models have been omitted for clarity.

Second, we focussed on a specific form of epilepsy linked to a limited set of genes. DS is one of the best described and genetically most homogeneous DEE syndromes.^27^ More than 80% of DS cases are attributable to variants in *SCN1A*^3^ (OMIM ID: 607208) and about 700 pathogenic CDS variants have been reported.^28^ Given the clear link between variants in *SCN1A* and DS, any un-annotated exonic sequence is of potential clinical interest. The updated *SCN1A* annotation identified nine exons, four shifted splice junctions and a 3′ UTR (Fig. 3; Table 1), increasing the genomic footprint of *SCN1A* transcription by ~3 kb (see Supplementary Fig. 1 for an illustration of all splicing features in Table 1 for *SCN1A*).

**Table 1:** List of all features identified within *SCN1A*.

Feature	Feature type	Feature length	Chr position (GRCh38)	Feature position	Transcript biotype	Transcript region	Feature conservation
1	Exon	168 nt	chr2:166,149,047–166,149,214	Terminal	Coding	5′ UTR	Yes
2	Exon	87 nt	chr2:166,126,924–166,127,010	Internal	Coding	5′ UTR	Partial - splice donor conserved
3	Exon	73 nt	chr2:166,126,982–166,127,055	Terminal	Coding	5′ UTR	No
4	Exon	111 nt	chr2:166,126,924–166,127,034	Terminal	Retained intron	5′ UTR	Yes
5	Splice acceptor	46 nt extension	chr2:166,077,802–166,077,848	Internal	Coding	5′ UTR	No
6	Exon	264 nt	chr2:166,071,623–166,071,886	Internal	Processed transcript	n/a	No
7	Exon	228 nt	chr2:166,060,640–166,060,867	Internal	NMD	CDS	No
8	Splice acceptor	4 nt extension	chr2:166,056,501–166,056,504	Internal	NMD	CDS	Yes
9	Exon	92 nt	chr2:166,053,039–166,053,130	Internal	Coding	CDS	Partial - splice acceptor conserved
10	Splice acceptor	3 nt truncation	chr2:166,045,325–166,045,327	Internal	Coding	CDS	Yes
11	Splice donor	16 nt extension	chr2:166,041,215–166,041,230	Internal	NMD	CDS	Yes
12	Exon	64 nt	chr2:166,007,230–166,007,293	Internal	NMD	CDS	Yes
13	Intron	Skips 282 nt exon	chr2:166,002,471–166,002,752	Internal	Coding	CDS	Yes
14	Exon	66 nt	chr2:165,999,051–165,999,116	Internal	NMD	CDS	No
15	Intron retention	Retains final intron of 1723 nt	chr2:165,992,413–165,994,147	Terminal	Coding	CDS	Yes

A single Ensembl transcript model is listed for all features; certain features are also present in other models. ‘Biotype’ details the functional effect of the feature as inferred by manual annotation. The alternative final exon within model ENST00000642141 was annotated as ‘non-coding’ due to the absence of polyadenylation data as per GENCODE guidelines; the functional status of this model is in reality unknown. Feature conservation describes the annotation and structurally identical feature in the mouse ortholog *Scn1a*

At least two of these annotations have demonstrably added biologically relevant sequences to the GENCODE catalogue. Firstly, we found that one exon had in fact already been reported in the human *SCN1A* literature, being described as alternatively spliced with respect to canonical exon 5 in a mutually exclusive manner^29^ (feature 9; Table 1, Fig. 3). The inclusion of this alternative exon is known to generate *SCN1A* isoforms that differ in their expression pattern (with the exon preferentially expressed in neonatal brain, as confirmed by our analysis) and sensitivity to the antiepileptic medications phenytoin and lamotrigine. Nonetheless, it appears that this exon had not previously been included in any gene annotation catalogues and we therefore presume it has also been absent from *SCN1A* exome panels. Secondly, a poison exon (feature 12) was also missing from GENCODE, despite the fact that the orthologous exon in rat has been experimentally characterised^30^ (feature 12; Table 1, Fig. 3). We report that this exon incorporates ClinVar variant RCV000209951, an *SCN1A* de novo variant found in a patient that was initially described as DS,^31^ but after re-examination of the phenotype, appeared to have febrile seizures plus: he had his first febrile seizure at the age of 23 months and later on developed afebrile tonic clonic seizures and febrile status epilepticus. He had some mild speech delay, but at the age of 11 years he had a normal development and was seizure free for 2 years.^32^ The variant had been annotated as intronic by ClinVar and thus of unknown significance. Genome alignments support the conservation of this exon across vertebrate species. This variant has since been independently characterised as a gain of function variant, promoting inclusion of the poison exon and leading to reduction in the amount of SCN1A protein.^32^

Two additional regions added to GENCODE, as part of this study, exhibit strong markers for functionality at the transcript level. The first is an alternative first exon consisting of 5′ UTR sequence, found ~21 kb upstream of the previously recognised 5′ end of *SCN1A* (feature 1; Table 1, Fig. 3). This exon was previously identified based on targeted sequencing of the locus,^33^ although it was absent in annotation catalogues until our work in this study. At the current time, there is limited evidence for an association of this exon with disease,^34^ although we suspect it remains largely unstudied in a clinical context. Certainly, the biological validity of the exon is underpinned by strong transcriptomics support in multiple data sources and also the fact that it is conserved in mammalian and avian genomes, with brain-specific transcriptomics support in mouse and chicken (not shown). The second region also represents additional 5′ UTR sequence, this time found as a cassette exon in the first intron following the ‘canonical’ *SCN1A* first exon (feature 2; Table 1, Fig. 3). Splice site conservation is observed across to avian genomes (although with a 3-bp acceptor site shift in certain lineages), with brain-specific RNA-Seq support in chicken (not shown). Neither of these two novel regions overlaps with any known disease-linked variants. However, as noted, it should be considered that these regions have apparently been hitherto unstudied in a clinical context.

**Fig. 4:**
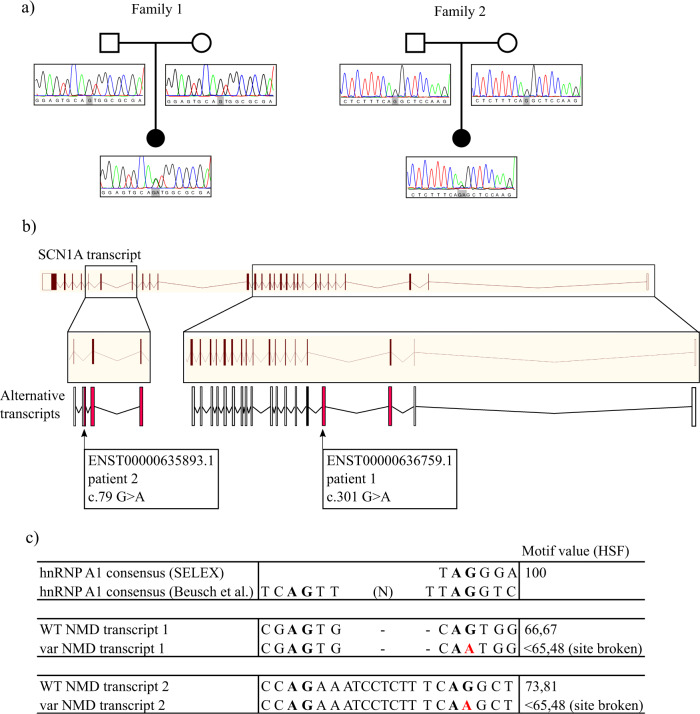
Variants in coding regions are associated with DS. **a** Pedigrees and Sanger sequencing traces of the two families with a de novo *SCN1A* variant in the identified poison exons. **b** The two transcripts containing the variants, relative to the full-length transcript. Red exons are coding, white exons are non-coding. **c** Variants are predicted to disrupt a hnRNP A1 recognition site.

To further investigate the value of our annotations in epilepsy diagnostics, we screened a cohort of 122 people with DS or a clinically similar phenotype for the added regions of *SCN1A*, plus seven other genes with known associations to this disorder (*SCN2A*, *SCN1B*, *GABRA1*, *GABRG2*, *HCN1*, *CHD2* and *PCDH19*). Our efforts in this study have increased the total transcript count of these genes from 56 to 135. Cohort inclusion criteria were (1) onset of seizures in the first years of life, (2) presence of some degree of developmental delay, (3) fever sensitivity and/or prominent myoclonic seizures, leading to inclusion of people with typical DS, but also clinically and genetically overlapping syndromes such as myoclonic astatic epilepsy. All people had previously undergone diagnostic genetic screening for variants in epilepsy-associated genes including *SCN1A*, but no clear causal variant had been identified. We identified two de novo variants in *SCN1A* in two people (Fig. 4).

Patient one is a 15-year-old girl diagnosed with DS. Previous screening efforts did not reveal a molecular diagnosis (including *SCN1A*, *STXBP1* and a gene panel^35^ consisting of known and candidate genes for DS and Myoclonic Atonic Epilepsy). The de novo variant identified here is found in an *SCN1A* poison exon (feature 7 originally in intron 1; GRCh38 chr2: 166060831, ENST00000636759.1:c.301 G > A; Table 1, Figs. 3 and 4). The variant was validated with Sanger sequencing and maternity and paternity was confirmed using an in-house developed multiplex PCR panel consisting of 16 STR-markers scattered over the genome, including the X and Y chromosomes. She is the only child of healthy non-consanguineous parents. The father had photosensitive epilepsy as a child but is now seizure-free without medication. A half-sister on the mother’s side has epilepsy but no developmental delay. Due to the normal development of both the father and the maternal half-sister and due to the lack of kinship between father and half-sister, it is not expected that the family members share the same genetic variant as the proband to explain their epilepsy. However, it cannot be excluded that the father has a low-grade mosaicism for the *SCN1A* variant that was not detected through Sanger sequencing. The proband first presented with febrile seizures when she was 8 months old. She had focal impaired awareness seizures and later also developed afebrile tonic-clonic seizures starting at 18 months old that occurred very frequently until the age of 5 years. She also had absences and myoclonic seizures. Electroencephalograms (EEG) showed background slowing and paroxysmal slow spike and spike waves. Development was normal prior to seizure onset, but slowed soon after, resulting in moderate intellectual disability. Brain MRI imaging showed no abnormalities and normal spectroscopic sequences. She is currently being treated with a combination of levetiracetam, topiramate, clobazam and stiripentol, but still has frequent tonic-clonic seizures.

Patient two is a 14-year-old girl diagnosed with DS. Previous screening of *SCN1A*, *PCDH19* and *HCN1* did not result in the identification of a pathogenic variant. Our study identified a de novo variant in a different poison exon of *SCN1A* (feature 14 originally in intron 22; GRCh38 chr2: 165999107, ENST00000635893.1:c.79 G > A; Table 1, Figs. 3 and 4). The variant was validated with Sanger sequencing and maternity and paternity confirmed using the same in-house developed multiplex PCR panel. She is born from healthy non-consanguineous parents. There is no familial history of epilepsy. The proband had her first febrile seizure evolving to status epilepticus when she was 6 months old. She further had, on average, six or seven episodes of generalised tonic-clonic status epilepticus per year, mostly with fever. After seizure onset her development slowed, resulting in moderate intellectual disability. Other comorbidities include ataxia, orofacial dyspraxia, difficulties with fine motor skills, hyperkinesia and sleep disturbances. EEG at the age of 5 years showed bifrontal slow waves and rare temporal spikes. MRI was normal apart from an asymptomatic pituitary cyst. She is currently being treated with a combination of valproate, clobazam and topiramate, which has reduced seizure frequency.

To quantify the significance of de novo variant enrichment in the 450 bp of CDS sequences (sum of features 7, 9, 12 and 14 from Table 1) and to estimate the probability of identifying two or more de novo variants in a cohort of 122 sequenced probands, we performed a de novo variant enrichment statistical test using the fitDNM package.^36^ This package returns the Poisson unweighted *P*-value based on expected mutability rate and shows that it is improbable to observe two de novo variants among 122 individuals along this stretch of 450 bp of CDS (unadjusted *p* = 9.5 × 10^−7^). Even after conservative exome-wide multiple testing correction for 18,000 possible protein-coding genes, this remains significant (*p* = 0.017), confirming a significant enrichment of de novo variants in the *SCN1A* sequences.

Neither poison exon is well conserved (PhastCons scores 0.102 and 0.000 for the two variants respectively). Both variants were predicted to alter binding sites for hnRNP A1, which is a splice ‘silencer’ that promotes exon skipping.^37,38^ We, therefore, postulate that the alterations of these respective motifs could lead to increased inclusion of the poison exons and therefore a reduction in the production of *SCN1A* protein due to NMD. Further functional work will be needed, however, to validate this hypothesis.

## Discussion

We have used human brain-transcriptomic data to rigorously interrogate the human reference transcriptome for missing transcriptional features, focusing on 191 genes associated with DEE. We investigated a cohort of people with DS, which has a robust phenotype/genotype correlation with *SCN1A*, with the presumption being that any such exon features that capture a variant in *SCN1A* could support a diagnosis. On investigation, we identified three de novo variants within three distinct poison exons of the *SCN1A* gene that are associated with DS, each of which is absent from current diagnostic tests. Many studies have underlined the important contribution of de novo variants in the aetiology of DEE,^2^ making such variants of particular interest to the clinician. Variants in poison exons have been previously described to cause Mendelian disorders, *via* mechanisms proposed to affect the level of protein expression.^39,40^ Haploinsufficiency of *SCN1A* is a cause of DS and it therefore seems reasonable to speculate that a higher inclusion of any of these poison exons could lead to a net reduction of functional protein and thus the disease phenotype; this has in fact now been established for ClinVar variant RCV000209951 in the poison exon described by Carvill et al.^32^ For the two de novo variants first reported here, we recognise that similar efforts to ascribe true pathogenicity will now be required, which is complicated by the neuron-specific expression pattern of *SCN1A*. However, given the strong and clearly established genotype–phenotype correlation between DS and *SCN1A*, it is appropriate to consider these two de novo variants as strong candidates for driving pathogenicity at the current time, not least because making this genetic diagnosis can underpin potentially life-saving changes to medication, as well as informing prognosis and stopping further unnecessary investigations. We emphasise that these are de novo variants and their absence in ~15,500 genomes in gnomAD^36^ is consistent with negative selection in human populations. Also, the observation of multiple disease-linked variants within the total 450-bp space of predicted coding or NMD-triggering sequence is highly improbable among just 122 probands - considering also that the average human is expected to have around 70 single nucleotide de novo variants in total^41^- which suggests they have been identified due to the ascertainment for a cohort of molecularly undiagnosed DS probands.

Nonetheless, it is striking that only the poison exon overlapping ClinVar variant RCV000209951 exhibits notable evolutionary conservation. This point is of immediate practical importance because the de novo variants reported here are at risk of being filtered out by prioritisation algorithms that utilise conservation metrics. These findings may also be surprising from a biological point of view, i.e. considering potential mechanisms of pathogenicity, given the traditional weight placed on the maxim that ‘conservation = function’. However, while gene-level conservation is typically studied in the context of protein-coding sequences, far less is known about the evolutionary dynamics of gene regulatory programmes linked to alternative splicing. These concepts may be especially relevant to the brain, which is known to be particularly rich in alternative splicing compared to other organs and tissues.^42^ Indeed, given that the human brain has evolved and enlarged considerably with respect to apes over the past 2.5 million years,^43^ we can speculate that functionality is generally linked to newly evolved sequences.^44^ Nonetheless, we also observed that the transcriptional support for both poison exons is not strong. These observations would be reconciled with our inferences into pathogenicity if the de novo variants do indeed turn out to lead to increased inclusion of the poison exons. It would therefore be informative to know whether these exons have higher levels of inclusion in the two DS people.

Extrapolating from this discussion, we consider that all of the 3550 transcripts annotated here within an exemplar set of 191 genes associated with epilepsy are of potential clinical interest. However, we recognise that detailed work will be required to establish which of these de novo variants have a true clinical association with epilepsy, as well as the biological mechanisms by which they drive disease. A logical next step would be to resequence these regions in people with epilepsy that lack a molecular diagnosis. In the meantime, it may be of interest to note that we also added two poison exons to *SCN8A* and five to *SCN2A*, which are recognised as brain-expressed sodium channel genes. These exons are apparently absent from other annotation catalogues or exome panels and we observe that four are conserved, at least across the mammalian order (GRCh38 chr12:51768113-51768172; chr12:51780202-51780271; chr2:165328404-165328538; chr2:165357774-165357857 (this feature will appear for the first time in GENCODEv33)).

We recognise that the value of adding large numbers of additional transcribed regions to disease-linked genes could be questioned, while it remains unclear exactly which transcript models have biological relevance. Broadly speaking, diagnostics based on expanded gene annotation has the potential to reduce false negative variant interpretations (i.e. to incorporate important ‘missed’ variants) at the expense of an increase in false positives. As discussed, our *SCN1A* variant interpretations benefit from the high concordance between perturbations to this gene and DS, as well as the fact that these are de novo variants. It is less certain how expanded annotation would perform in clinical scenarios where this is not the case. For example, hundreds of genes have now been linked to autism spectrum disorder with varying degrees of confidence and this disorder has a far more heterogeneous causation than DS.^42^ In our view, identified sequences with strong markers for functionality – especially those that can be established as coding sequences based on evolutionary conservation and/or proteomics data – should be considered those most likely to have functionality and therefore those with the most potential value in the search for undiscovered disease-linked variants. Nonetheless, our work here illustrates that a consideration of the full transcriptional profile of a gene can also be fruitful, i.e. including transcripts with poor conservation and weak transcription and those that do not appear to encode proteins. As discussed, we believe this point is particularly apposite when considering that pathogenicity can also arise from gain of function modes.

Finally, establishing a genetic cause for epilepsy in an individual is a key step in clinical management. It provides an explanation, terminates the diagnostic odyssey and may inform treatment options and prognostication. Furthermore, it helps with determining other management issues (e.g. known associated comorbidities like gait disorders, involvement of other organs, dysphagia), genetic counselling and overall care, where having a name for a condition typically facilitates access to services. Importantly, it also provides a label and typically relieves parental guilt. Last, but not least, identifying the genetic cause of a severe disease can have direct therapeutic implications, like avoiding the use of sodium channel blockers, or using stiripentol as add-on therapy,^45^ in DS caused by *SCN1A* variants. Current methods of establishing a genetic diagnosis in clinical settings consist of candidate gene analysis, gene panel or WES and array comparative genomic hybridisation to identify copy number variants (CNVs). If these methods are undertaken and no variant is found, such cases usually go into research projects, which generally take longer and are more uncertain. If the original tests to establish a diagnosis are missing annotation, this all leads to costly and unnecessary delays, both for patients, their family and for the healthcare system. This study further raises the question whether it would be more desirable to use WGS for diagnostic purposes, as data can be iteratively re-annotated when updated annotation information becomes available. Similarly, this approach has already been proven successful as iteratively re-analysing patient genomes when new causative genes are discovered increases diagnostic yield.^7,46^

In summary, our findings suggest that there are potentially additional causative genetic variants to be identified in and around epilepsy-related genes such as *SCN1A*, including in predicted poison exons. We anticipate that the inclusion of the transcripts identified here will further increase the number of variants found in *SCN1A* in people with DS. Furthermore, if the same approach we have taken to *SCN1A* and DS is applied to other focussed cohorts for the other 190 genes in our study, we would expect to find resolvable cases in the gene that was originally suspected by the clinician.

## Methods

### Selection of genes

191 genes associated with deleterious variants implicated in epilepsy in general and DEE in particular were included in this study. These included 66 genes from the Great Ormond Street Hospital (GOSH) for Children, London, UK, Early Infantile Epileptic Encephalopathy (EIEE) gene-panel (http://www.labs.gosh.nhs.uk/media/759010/eiee_v7.pdf), selected as established causes of early-onset seizures and/or severe developmental delay in patients without frequent major structural brain anomalies. Genes leading to neurometabolic disorders with readily identifiable blood/urine/cerebrospinal fluid (CSF) biomarkers were not included. An additional 58 genes were included from the Addenbrooke’s Hospital, Cambridge, UK, EIEE gene panel, as well as 14 genes from the DDD study^47^ and 53 genes from literature searching. The full list can be found in Supplementary Data 2.

### Gene annotation

Manual re-annotation of the 191 genes was performed on GRCh38 (https://www.ncbi.nlm.nih.gov/grc/human) according to the guidelines of the HAVANA (Human And Vertebrate Analysis and Annotation) group^48^ (and ftp://ftp.sanger.ac.uk/pub/project/havana/Guidelines/Guidelines_March_2016.pdf). In summary, the HAVANA group produces annotation, largely based on the alignment of transcriptomic (ESTs and mRNAs) and protein sequence data from GenBank^49^ and Uniprot.^50^ These data were aligned to the individual bacterial artificial chromosome clones that make up the reference genome sequence using BLAST^51^ with a subsequent realignment of transcript data by Est2Genome.^52^ In addition, SLR-RNA-Seq data^53^ mapped using Gmap,^54^ PACBIO^55^ reads mapped using STAR^56^ and foetal and infant RNA-Seq data^19^ mapped using cufflinks,^57^ were also used to identify transcripts and splice junctions. Data are available at www.gencodegenes.org/releases/. Updated annotation of the 191 genes described in this study on GRCh38 is represented in GENCODE releases from v27 (August 2017) onwards. In addition, the updated annotation is available remapped to GRCh37 [https://github.com/diekhans/gencode-backmap] here: http://www.gencodegenes.org/releases/grch37_mapped_releases.html.

### Identification of splicing events

Transcript structures in public releases of GENCODE before (GENCODE v20) and after (GENCODE v28) the manually updated annotations were compared to find exons, introns and shifted splice junctions. The number of genomic bases covered by the extended gene annotation and coding sequence was calculated using a custom Perl script. Exons are defined as those in the updated annotation that shared no sequence with any exon in the previous annotation. Introns were those introns in the updated annotation that did not match exactly an intron in the old annotation. Shifted splice junctions occurred when an exon in the updated annotation shares an overlap with an exon in the old annotation but at least one of the splice sites was not in the same location. Retained intron transcripts were excluded from this analysis. RefSeq annotation for transcript counting was extracted from the Ensembl release 84 (March 2016) “RefSeq GFF3 annotation import”.

### Identification of transcriptional features on GRCh38 using foetal and infant RNA-Seq data

Illumina data from Jaffe et al.^19^ was re-mapped for foetal and infant transcriptome to GRCh38 to identify transcriptional features. FASTQ files from the following datasets were downloaded from ENA: SRR1554537, SRR1554538, SRR1554541, SRR1554544, SRR1554546, SRR1554549, SRR1554551, SRR1554553, SRR1554554, SRR1554566, SRR1554567 and SRR1554568. Data were mapped to GRCh38 with TopHat (tophat-2.0.13).^58^ Reads mapping to the gene regions that were studied, were merged into two files containing foetal and infant alignments using SAMtools.^59^ Transcript models were generated from the foetal and infant BAM files using Cufflinks (cufflinks-2.2.1).^58^ Introns and splice sites were identified, a BED file generated and passed to manual annotators for checking.

### Quantification of gene expression at exon level

Raw reads from Jaffe et al.,^19^ were available via study accession SRP045638. The 36 paired-end libraries were analysed using the iRAP pipeline (https://github.com/nunofonseca/irap). First, raw reads in the original FASTQ files underwent quality assessment and filtering.^60^ They were then aligned against the GRCh38 genome reference using Tophat2,^61^ with the option: ‘–min-intron-length 6’.

### Analysis of expression of splice features

Integrative Pipeline for Splicing Analyses (IPSA)^62^ was employed to produce splice junctions and their read counts from Tophat2 alignments of Jaffe et al.^19^ on GRCh38 human genome. This analysis included 36 human brain pre-frontal cortex samples, corresponding to six different developmental stages (Foetal, Infant, Child, Teen, Adult and Old).^19^ IPSA was run with the default parameters and the pre-annotation update release GENCODE v20 as a reference. Transcript expression levels around introns were estimated from the number of reads supporting the respective splice junction. Expression of splice junctions was normalised by the total number of reads in each sample.

### Mapping annotation from reference human genome to mouse genome

The TransMap cross-species alignment algorithm was used to map all annotated transcripts from the reference human genome (GRCh38) to the reference mouse genome (GRCm38). The alignments are created using synteny-filtered pairwise genome alignments (chains and nets) produced using BLASTZ.^23,63,64^ All transcript models mapped to mouse were manually-assessed to identify failures to align correctly at the base, exon and intron level.

### Identification of conservation of coding sequence

The DEE gene annotation was obtained by subtracting the GENCODE v20 gene annotation from the current one (equivalent to GENCODE v28) using “bedtools subtract” separately for exon and CDS regions. The RepeatMasker repeat features (except low complexity elements) extracted from Ensembl were subsequently subtracted from this annotation. The filtered annotation was then intersected with:

- phastConsElements100way.bed obtained from the UCSC Table Browser (https://genome.ucsc.edu/cgi-bin/hgTables);
- 27 amniota vertebrates GERP constrained elements from Ensembl (ftp://ftp.ensembl.org/pub/release-90/bed/ensembl-compara/27_amniota_vertebrates_gerp_constrained_elements/gerp_constrained_elements.homo_sapiens.bed.gz);
- PhyloCSF (58 mammals) approximate coding regions from the PhyloCSF track hub (https://data.broadinstitute.org/compbio1/PhyloCSFtracks/trackHub/hg38/trackDb.txt).

In all cases, the overlap with the DEE gene annotation was carried out using “bedtools intersect”.

### Identification of variants in updated annotation

The collection of variants available under “All phenotype-associated - short variants (SNPs and indels)” in Ensembl release 90 (August 2017) was intersected separately with the exons in GENCODE 20 and in the current annotation, using a custom Perl script and the Ensembl API. The variants overlapping the current annotation, but not GENCODE 20, were reported. A second round was carried out, considering only the 8-nt exon flanking sequences in both annotation sets.

As a proof-of-concept, we screened a cohort of 122 people with DS or a clinically similar severe myoclonic epilepsy phenotype for the 125 regions identified from this study of all genes that have previously been associated with DS (*SCN1A*, *SCN2A*, *SCN1B*, *GABRA1*, *GABRG2*, *HCN1*, *CHD2* and *PCDH19*), using an amplicon targeted amplification assay (Agilent, https://www.agilent.com). All samples underwent diagnostic screening for *SCN1A* (including both sequencing and CNV analysis), but no pathogenic variants had been identified, after which they were included in research. Additionally, several patients were screened for genetic variants in epilepsy-associated genes using Sanger sequencing, gene panels or WES.

Primers for the multi-amplicon target panel were designed using the mPCR software (Agilent).^65^ Specific target regions were amplified using multiplex PCR, followed by purification of the equimolar pooled amplicons using Agencourt AMPureXP beads (Beckman Coulter, CA, USA). Individual barcodes (Illumina Nextera XT) were incorporated in a universal PCR step prior to sample pooling. Libraries were sequenced on a MiSeq platform using v3 reagent kit with a paired-end read length of 300 bp (Illumina, USA).

Analysis was performed in-house with a standardised pipeline integrated in genomecomb.^66^ The pipeline used fastq-mcf (https://expressionanalysis.github.io/ea-utils/) for adapter clipping. Reads were then aligned using BWA-MEM^67^ and the resulting SAM file converted to BAM using Samtools.^59^ BAM files were sorted using biobambam.^68^ Realignment in the neighbourhood of indels was performed with GATK.^69^ Amplicon primers were clipped using genomecomb.^66^ Variants were called at all positions with a total coverage ≥5 using both GATK^69^ and Samtools.^59^ At this initial stage, positions with a coverage <5 or a score <30 were considered unsequenced. The resulting variant sets of different individuals were combined and annotated and filtered using genomecomb.^66^

Variants with a coverage above seven and a GATK quality score above 50 that were identified by both variant callers (GATK and Samtools) and were absent in publicly available databases (gnomAD,^70^ 1000 Genomes,^71^ Exome Variant Server (http://evs.gs.washington.edu/EVS/)) and our in house database, were extracted. Furthermore, variants present in homopolymer regions (>8 homopolymers) or simple repeat regions were excluded. The effect of the variant was predicted with the Ensembl Variant Effect Predictor (VEP),^72^ using the manually annotated GENCODE dataset as custom gene annotation. Variants were validated and the segregation was checked using bi-directional Sanger sequencing. For de novo variants, maternity and paternity was confirmed using an in-house developed multiplex PCR panel consisting of 16 STR-markers scattered over the genome, including the X and Y chromosome.

To predict the effect of the de novo variants on splicing efficiency, we used the ‘quick mutant’ analysis from Human Splicing Finder,^73^ using the CDS from the NMD transcripts as custom sequence input.

### Testing for significant excess of de novo variation among *SCN1A* CDS protein-coding sequence

The genomic sequences corresponding to protein-coding features of *SCN1A* (Features 7, 9, 12 and 14; Table 1) were concatenated to reflect a consecutive test sequence of length 450 bp on GRCh38. To test whether the observed number of two de novo variants among this stretch of 450 bp was significant, based on a sampled cohort of 122 individuals, we adopted a modified version of R package fitDNM.^36^ The published fitDNM provides both a PolyPhen-2 weighted and Poisson unweighted *p*-value. Here, since PolyPhen-2 scores do not exist for the entirety of the CDS sequence we focus on the Poisson unweighted *P*-value. The modification to the original fitDNM package was to correct a type conversion error, which possibly occurred due to different versions of R used for our analysis (v3.5.0) compared to the original package version. Specifically, we fixed a clash where a ‘T’-valued variable (intended for Thymine) was handled as “TRUE”. The adapted fitDNM package accompanied by input and output files from this analysis are available upon request. This R package then takes as input the underlying mutability of the 450 bp (Supplementary Data 3), the total number of observed de novo variants among that 450 bases of sequence and the total number of probands tested for a de novo variant in that 450 bases of sequence. Subsequently, we conservatively corrected the resulting Poisson unweighted P-value by 18,000 to reflect approximately the total number of WES studied protein-coding genes in the human exome.

### Reporting summary

Further information on research design is available in the Nature Research Reporting Summary linked to this article.

## Supplementary information

Supplementary Information

Supplementary Data 1

Supplementary Data 2

Supplementary Data 3

Reporting Summary Checklist

## Data Availability

All data are available from the authors. Transcript annotation is available to download from https://www.gencodegenes.org/human/. Both variants are available from ClinVar under accession numbers SCV000995824.1 and SCV000995825.1.
