# Supplementary material for: Re-annotation of 191 developmental and epileptic encephalopathy-associated genes unmasks de novo variants in SCN1A
Source: NPJ Genom Med. 2019 Dec 2;4:31. doi: 10.1038/s41525-019-0106-7 (PMC6889285; doi:10.1038/s41525-019-0106-7)
Supplement: Supplementary file 1 — Supplementary Information [file 41525_2019_106_MOESM1_ESM.pdf]

Supplementary Data 1

The first sheet lists the 23 variants found in the ClinVar database that are incorporated into the exon annotations produced in this study. Additional variants found within 8bp of existing splice sites were filtered out and are presented in sheet 2. Note that ClinVar is primarily based on RefSeq annotation; most of the variants in the table are still considered as intergenic or intragenic by ClinVar and the classifications of pathogenicity presented there will not necessarily change according to these annotations. Genome coordinates are for GRCh38.

Supplementary Figure 1

Figure showing the position of all features identified within *SCN1A* as viewed in the Genoverse browser <https://wtsi-web.github.io/Genoverse/>). The 5' end is to the right and the 3' end is to the left. See Table 1 in the main manuscript for a description of each of the 15 identified features. Panel A below shows the entirety of *SCN1A* and the 15 features, which are illustrated with arrows. Panels B-E show a higher resolution of these features starting at the 5' UTR (panel B) and ending with the 3' UTR (panel E).

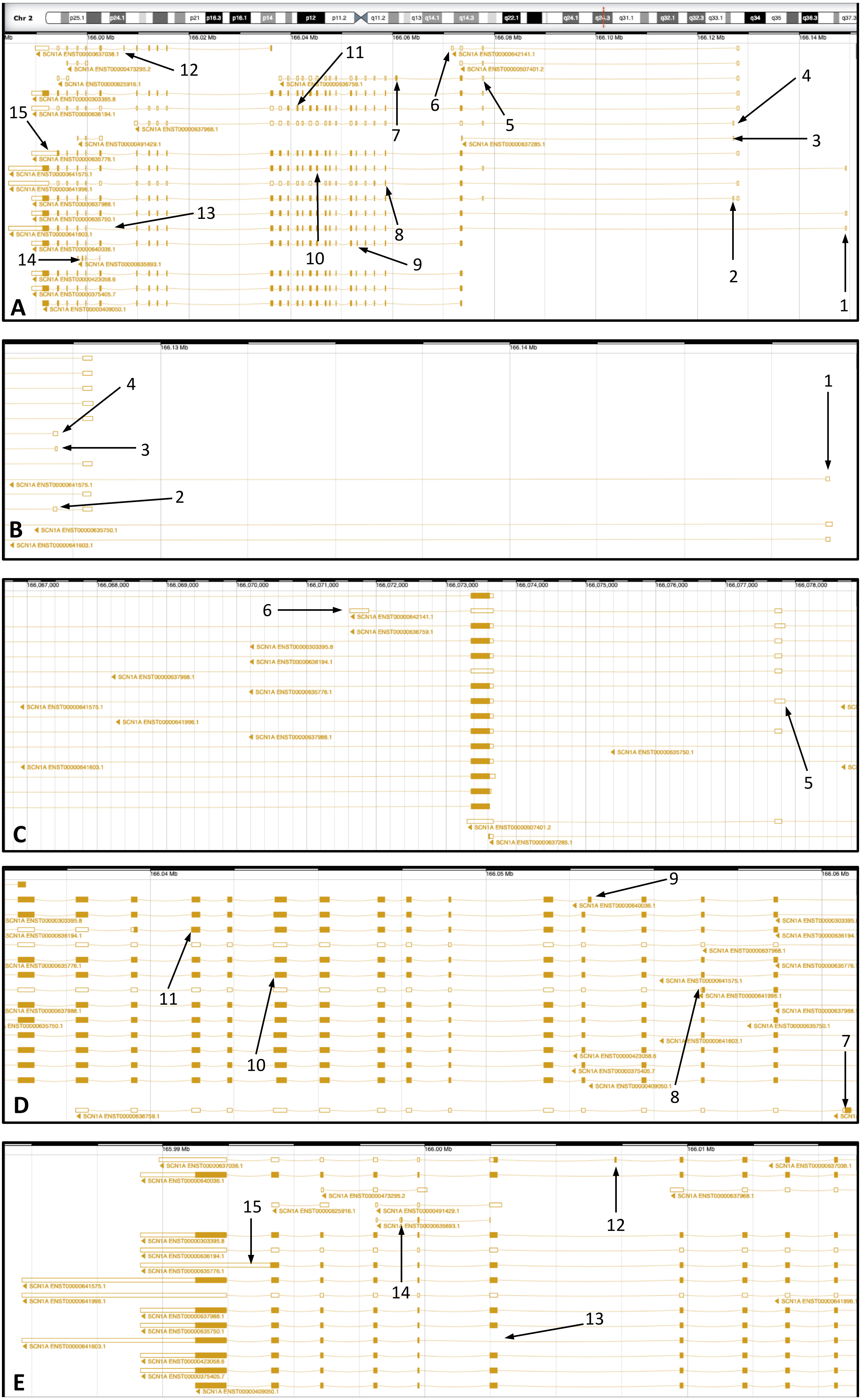
Chr 2
SCN1A ENST00000637038.1
SCN1A ENST00000473295.2
SCN1A ENST00000625916.1
SCN1A ENST00000303395.8
SCN1A ENST00000636194.1
SCN1A ENST00000637968.1
SCN1A ENST00000491429.1
SCN1A ENST00000635776.1
SCN1A ENST00000641575.1
SCN1A ENST00000641996.1
SCN1A ENST00000637988.1
SCN1A ENST00000635750.1
SCN1A ENST00000641603.1
SCN1A ENST00000640036.1
SCN1A ENST00000635893.1
SCN1A ENST00000423058.6
SCN1A ENST00000375405.7
SCN1A ENST00000409050.1
SCN1A ENST00000642141.1
SCN1A ENST00000507401.2
SCN1A ENST00000636759.1
SCN1A ENST00000637285.1
A
B
C
D
E

Supplementary Data 2

The complete list of Ensembl gene IDs and HGNC gene names (https://www.genenames.org/) that were reannotated as part of the study. Also listed is the number of number of transcripts found before reannotation (GENCODE v20 transcripts) and after reannotation (GENCODE v28 transcripts). The Mendelian Inheritance in Man accession ID for each gene is listed in the final column (https://www.omim.org/)

Supplementary Data 3

Results from the fitDNM package analysis across the 450bp of CDS from exons 7,9,12 and 14 in *SCN1A*. Refer to Table 1 in the main manuscript.
